# The Interaction of Genetic Mutations in PARK2 and FA2H Causes a Novel Phenotype in a Case of Childhood-Onset Movement Disorder

**DOI:** 10.3389/fneur.2019.00555

**Published:** 2019-05-29

**Authors:** Matthew Benger, Kshitij Mankad, Christos Proukakis, Nicholas D. Mazarakis, Maria Kinali

**Affiliations:** ^1^Department of Neurosciences, King's College Hospital, London, United Kingdom; ^2^Department of Neuroradiology, Great Ormond Street Hospital, London, United Kingdom; ^3^Institute of Neurology, University College London, London, United Kingdom; ^4^Centre for Neuroinflammation & Neurodegeneration, Imperial College, London, United Kingdom; ^5^Honorary Senior Lecturer in Paediatric Neurology, Imperial College, London, United Kingdom

**Keywords:** PARK2 mutation, movement disorder, hereditary spastic paraplegia (HSP), novel phenotype, FA2H gene

## Abstract

Mutations in the *PARK2* gene have been implicated in the pathogenesis of early-onset Parkinson's disease. We present a case of movement disorder in a 4-year-old child from consanguineous parents and with a family history of Dopamine responsive dystonia, who was diagnosed with early-onset Parkinson's disease based on initial identification of a pathogenic *PARK2* mutation. However, the evolution of the child's clinical picture was unusually rapid, with a preponderance of pyramidal rather than extrapyramidal symptoms, leading to re-investigation of the case with further imaging and genetic sequencing. Interestingly, a second homozygous mutation in the *FA2H* gene, implicated in Hereditary spastic paraplegia, was revealed, appearing to have contributed to the novel phenotype observed, and highlighting a potential interaction between the two mutated genes.

## Case Background

A 4-year-old right-handed boy from Qatar presented with a 1-year history of increasingly frequent falls and progressive gait abnormality. Pregnancy, birth, and neonatal development all proceeded unremarkably. He sat independently by 6 months and walked by 13 months, although his parents noted he did so “heavily” and on his tiptoes bilaterally. He was able to jump but not hop, and negotiated staircases by holding onto the banisters. The parents had no concerns regarding handwriting, cognition, behavior, vision, or hearing. Finally, no diurnal variation in the symptoms had been noted.

The patient was one of three children born to healthy first cousins. His 3-year-old brother and 18-month-old sister were both asymptomatic. His father's 35-year-old brother presented with dystonia aged 13 years. Although his genotype was untested, he was given a clinical diagnosis of Dopamine-responsive dystonia (DRD), which remains Levodopa-responsive today.

**Figure 1:**
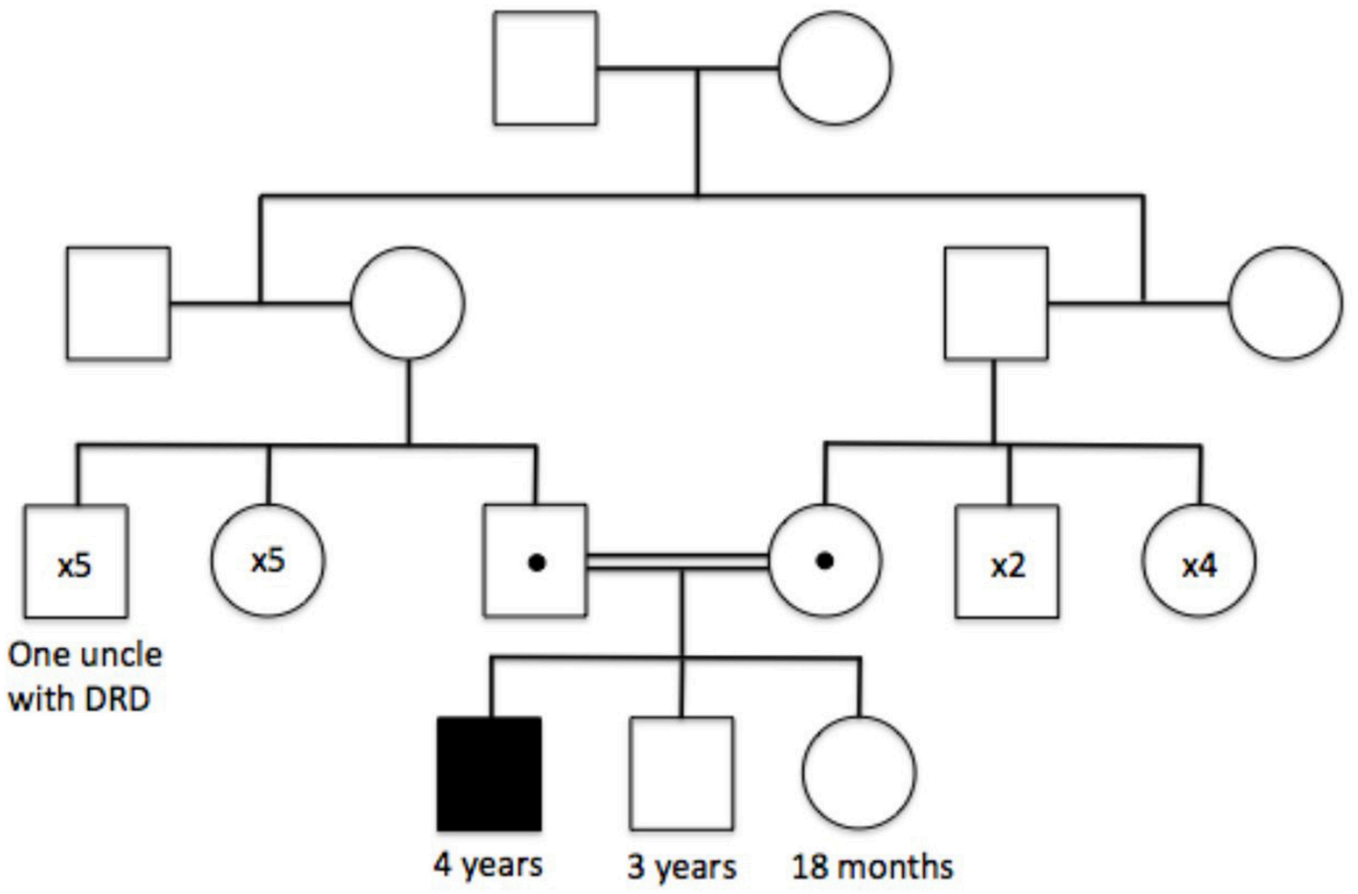
Family tree illustrating the autosomal recessive inheritance of the *PARK2* p.Ala138Glyfs^*^7 mutation, and the clinically affected family members at the time of initial genetic testing.

The patient had already received a number of tests in Qatar. These included an MRI scan 6 months prior to presentation (reported as normal) and genetic sequencing of *PARK2*, a gene well known to cause early-onset Parkinson's disease (EO-PD) (1). Regarding the latter, PCR amplification of exon 4 of *PARK2* failed after several attempts, and subsequent MPLA analysis confirmed a homozygous, pathogenic deletion in exon 4 of the *PARK2* gene (p.Ala138Glyfs^*^7), leading to a diagnosis of EO-PD, with both parents subsequently identified as being carriers of the same mutation (Figure 1). However, the patient underwent two empirical trials of Levodopa without benefit, prompting the parents to seek a second neurological opinion.

Examination (Video 1 in Supplementary Material) revealed a markedly paretic gait, with the patient walking, without festination or freezing, on his tiptoes with hips internally rotated in a crouched posture. There was some dystonic posturing of his limbs when lying on the couch. Both lower limbs exhibited hypertonia and hyperreflexia with clonus and upgoing plantars. His upper limbs were less affected, demonstrating subtle rigidity, more pronounced on the left. There was no tremor and no micrographia. There was subtle hypomimia. Cranial nerve examination was normal. The patient followed instructions with minor hesitation and interacted with his siblings normally.

In summary, the clinical picture was predominantly an upper motor neuron-type deficit affecting the patient's lower limbs. In addition to lower limb pyramidal features, there were less pronounced extrapyramidal features including dystonia in the lower limbs, asymmetrical rigidity in the upper limbs, and hypomimia. Hence, this was an early-onset, progressive neurological disorder affecting multiple motor systems. The predominance of pyramidal features, as well as the lack of Levodopa response, argued against this being a disorder driven purely by mutated *PARK2*.

## Diagnostic Investigations

**Figure 2:**
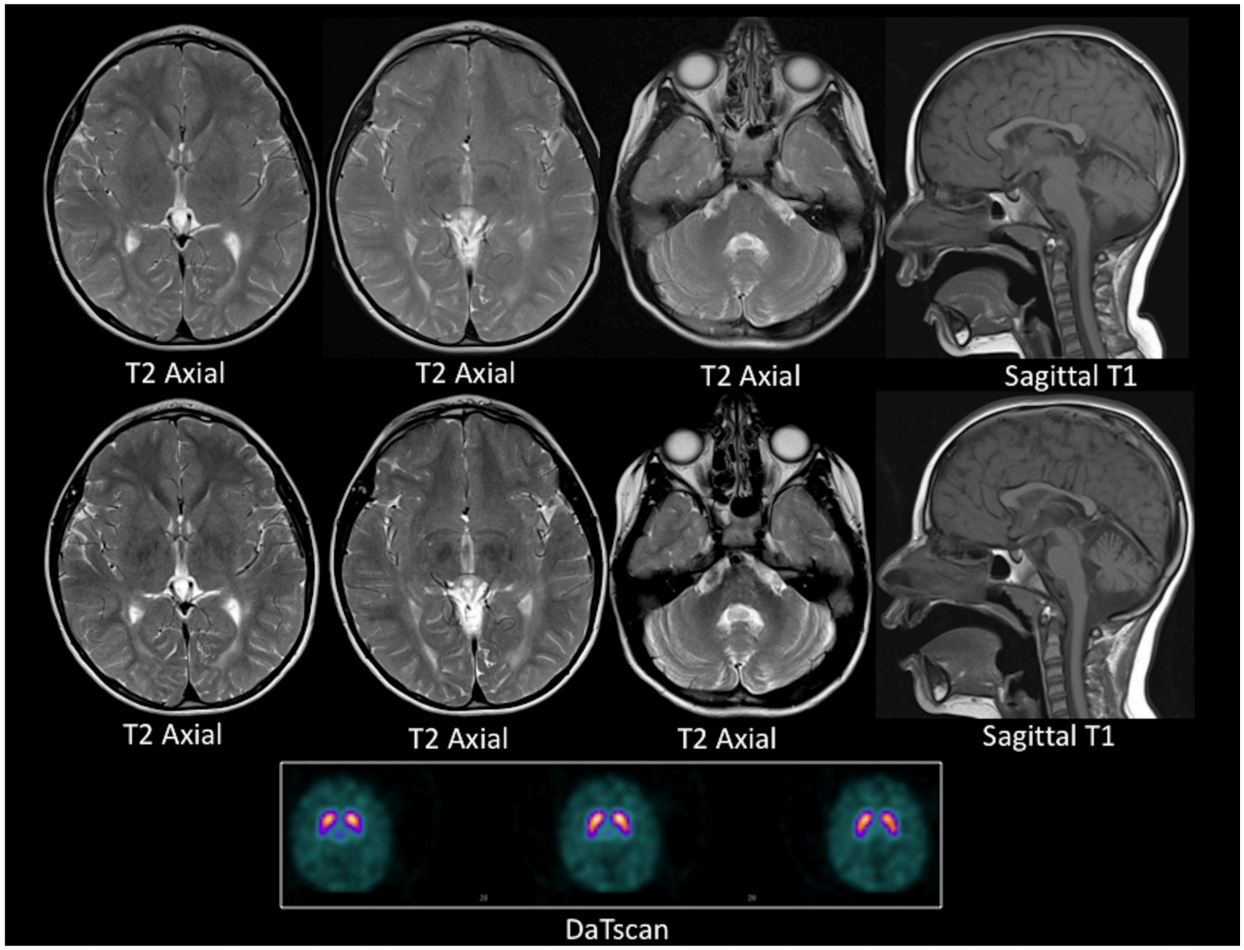
MRI and DaTSCAN images. Top row: Axial T2 weighted images and Sagittal T1 weighted image at age 4 years. Middle row: Axial T2 weighted images Sagittal T1 weighted image at age 5 years. Lower panel: DaTSCAN. All MR images were acquired on a 1.5T scanner. The top and the middle row images show progressive iron deposition in the globus pallidi, more than expected for age, and consistent with neuronal brain iron accumulation. There is global paucity of the cerebral white matter with a thin corpus callosum. There is also cerebellar atrophy between the two time points. The DaTSCAN is normal.

A repeat brain and spine MRI scan was performed under general anesthetic. In contrast to the previous scan, this demonstrated mildly increased T2 signal intensity in the deep and periventricular white matter, predominantly in the peritrigonal region of the lateral ventricles and the occipital region of the cortex, consistent with leukodystrophy. In addition, the repeat MRI demonstrated mild cerebellar volume loss, and bilateral symmetrical iron deposition within the substantiae nigrae and globus pallidi. These radiological changes progressed over a one-year interval (Figure 2). A Dopamine Transporter scan (DaTSCAN) was also carried out at the time of presentation and was reported as normal (Figure 2).

A raft of neurometabolic tests were negative, including CSF studies for neurotransmitters, pyridoxal phosphate and pterin metabolites. Given the patient's spastic paraparetic phenotype, and the above MRI findings, an additional diagnosis of hereditary spastic paraplegia (HSP) was considered.

DNA mutation analyses of a panel of HSP genes revealed a homozygous, glycine to alanine substitution (c.786+1G>A) following exon 5 of *Fatty acid 2-hydroxylase* (*FA2H*), a gene responsible for the hydroxylation of myelin galactocerebroside lipid components. *In silico* analysis of this mutation using Human Splicing Finder software (URL: http://www.umd.be/HSF3/, accessed on 04/04/2019) predicted it would alter an essential “GU” splicing donor sequence of wild type FA2H and should thus be considered pathogenic (2). Mutation of *FA2H* leads to HSP35 (also known as SPG35), a disorder characterized by childhood-onset spastic paraparesis (±dystonia, ataxia, seizures, progressive cognitive decline) (3, 4) and T2 signal hyperintensities on MRI ± brain iron accumulation (5, 6).

## Management

Treatments for HSP are currently limited to those providing symptomatic benefit, alongside genetic counseling for the family. Regular patient follow-up is also crucial, not only to monitor response to symptomatic treatments, but also to identify development of any of the clinically heterogeneous features of HSP35. Furthermore, in this patient's case, whilst the negative DaTSCAN would argue against mutated *PARK2*-related extrapyramidal features at time of presentation (which were instead more likely to be caused by iron accumulation in the basal ganglia secondary to mutated *FA2H*), it would be important to continue to monitor for manifestation of mutated *PARK2*. Indeed, 18 months after the initial presentation, worsening upper limb rigidity was noted, along with bradykinesia, freezing effect, and marked hypomimia, without significant changes in brain iron accumulation on repeat MRI, prompting consideration of a repeat DaTSCAN and levodopa trial at time of writing.

## Discussion

Ockham's razor, a frequently cited diagnostic tool, argues that “plurality should never be posited without necessity (7)”. In the case above, the simplest explanation for the patient's symptoms—an altered gait and a propensity to fall—would have pointed to the patient's known recessive *PARK2* mutation.

However, there were elements within the history and examination—particularly the pyramidal features and lack of dopamine responsiveness—which could not be “shaved away” for the sake of simplicity. Such features, along with symptom progression, justified the repeat MRI scan, which held the key to diagnosing the additional pathogenic *FA2H* mutation. The phenotypic overlap demonstrated by this patient in relation to the key clinical features of *PARK2*-related EO-PD and HSP35 is outlined in Table 1 (9–12).

**Table 1:** Clinical features of *PARK2-associated* EO-PO and HSP35 and comparison to the patient studied (8–11).

	***PARK2-associated* EO- PD**	**HSP35**	**Patient**
Mutation	Pathogenic mutations in both alleles of *PARK2*	Pathogenic mutations in both alleles of *FA2H*	Homozygous mutations of *FA2H* and *PARK2*
Age of onset	Typically 20–40 years	Childhood	Motor abnormalities noted at ~13 months
Progression	Slowly-progressive (typically over decades)	Variable but often rapid (frequently wheelchair-dependent by early adulthood)	Rapid deterioration over months
Dopamine responsiveness	Exquisite response	No response	No response
Pyramidal features	Frequently have hyperreflexia	Lower limb spasticity and weakness with hyperreflexia, clonus, and upward plantars	Lower limb spasticity and weakness with hyperreflexia, clonus, and upward plantars
Extrapyramidal features	Parkinsonian features including rigidity, tremor, bradykinesia, and hypomimia. Dystonia and postural instability and frequently prominent.	Frequently have dystonia	Dystonia in the lower limbs, asymmetrical rigidity in the upper limbs, and hypomimia
Other distinguishing features	Less prevalent versus sporadic PD. Anxiety may feature.	lntellectuaI decline, seizures, optic atrophy	None clearly identified
MRI findings	Often normal in early disease	Periventricular white matter T2 signal hyperintensities, cerebellar atrophy, iron accumulation in the basal ganglia	Periventricular white matter T2 signal hyperintensities, cerebellar atrophy, iron accumulation in the basal ganglia

*The patient demonstrates an overlapping phenotype with clinical features associated with both genetic mutations. Of note, the patient's Parkinsonian phenotype appears earlier than is typical for EO-PD, whilst the rate of clinical deterioration appears to be faster than is typically encountered with either EO-PD or HSP35 individuals, perhaps suggesting compound toxicity of the two genetic mutations (see discussion)*.

An additional point of interest remains the genotype of the patient's uncle, who was clinically diagnosed with DSD aged 13 without genotypic confirmation. An intriguing possibility remains that the patient's uncle in fact had homozygous mutation of the *PARK2* gene and was demonstrating features of undiagnosed EO-PD (presenting closer to a typical age of onset than in our patient). Certainly, there is considerable phenotypic overlap between DRD and PARK2-related EO-PD (7, 13), alongside the possibility of *PARK2* mutations on both maternal and paternal branches of the uncle's family tree due to undocumented consanguinity in previous generations.

At the genetic level, dual homozygous mutations in *PARK2* and *FA2H* have not been reported previously in EO-PD. Both genes play a role in mitochondrial function (a key organelle in the regulation of cellular toxicity and death): the former via a well-established role in optimal mitophagy, the latter via recently discovered links between ceramide metabolism, mitochondrial outer membrane permeability, and electron transport chain activity (14–18). Dual mutation may thus engender additional, or even synergistic, toxicity within nigrostriatal neurons, which might lead to an earlier-than-expected age of symptom onset (typically ~20–40 years) (12, 19). Indeed, whilst the patient's initial DaTSCAN was negative, he went on to develop progressive parkinsonism without corresponding changes in brain iron accumulation on interval MRI suggesting clinical progression of the patient's mutated *PARK2*-associated phenotype, and prompting consideration of a repeat DaTSCAN and Levodopa trial.

## Informed Consent

Written informed consent was obtained from the parents of the participant for the publication of this case report, and from all subjects featured in video material.

## Author Contributions

MB: composed manuscript. KM: reported case imaging and selected relevant images formanuscript. CP and NM: aided literature search and edited manuscript. MK: consultant clinician for the case, aided literature search, and edited manuscript.

### Conflict of Interest Statement

The authors declare that the research was conducted in the absence of any commercial or financial relationships that could be construed as a potential conflict of interest.

## Supplementary Material

Video 1 Demonstration of patient's gait at time of presentation. Marked lower limb spasticity is noted without festination or freezing. The patient walks on his tiptoes with hips internally rotated.
